# Red Sea corals under Artificial Light Pollution at Night (ALAN) undergo oxidative stress and photosynthetic impairment

**DOI:** 10.1111/gcb.14795

**Published:** 2019-09-11

**Authors:** Inbal Ayalon, Laura F. de Barros Marangoni, Jennifer I. C. Benichou, Dror Avisar, Oren Levy

**Affiliations:** ^1^ Mina and Everard Goodman Faculty of Life Sciences Bar‐Ilan University Ramat Gan Israel; ^2^ Israel The H. Steinitz Marine Biology Laboratory The Interuniversity Institute for Marine Sciences of Eilat Eilat Israel; ^3^ Porter School of the Environment and Earth Sciences Faculty of Exact Sciences Tel Aviv University Tel Aviv Israel

**Keywords:** ALAN, corals, light pollution, photosynthesis, physiology, ROS

## Abstract

Coral reefs represent the most diverse marine ecosystem on the planet, yet they are undergoing an unprecedented decline due to a combination of increasing global and local stressors. Despite the wealth of research investigating these stressors, Artificial Light Pollution at Night (ALAN) or “ecological light pollution” represents an emerging threat that has received little attention in the context of coral reefs, despite the potential of disrupting the chronobiology, physiology, behavior, and other biological processes of coral reef organisms. Scleractinian corals, the framework builders of coral reefs, depend on lunar illumination cues to synchronize their biological rhythms such as behavior, reproduction and physiology. While, light pollution (POL) may mask and lead de‐synchronization of these biological rhythms process. To reveal if ALAN impacts coral physiology, we have studied two coral species, *Acropora eurystoma* and *Pocillopora damicornis*, from the Gulf of Eilat/Aqaba, Red Sea, which is undergoing urban development that has led to severe POL at night. Our two experimental design data revealed that corals exposed to ALAN face an oxidative stress condition, show lower photosynthesis performances measured by electron transport rate (ETR), as well as changes in chlorophyll and algae density parameters. Testing different lights such as Blue LED and White LED spectrum showed more extreme impact in comparison to Yellow LEDs on coral physiology. The finding of this work sheds light on the emerging threat of POL and the impacts on the biology and ecology of Scleractinian corals, and will help to formulate specific management implementations to mitigate its potentially harmful impacts.

## Graphical abstract

The illustration shows how Artificial Light Pollution at Night (ALAN) can impact coral reefs by causing different physiological responses leading to photosynthetic performances and increasing oxidative stress.

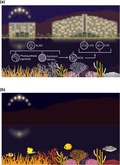

## 1 INTRODUCTION

Natural illumination at night is derived from the moon, the stars, and the Milky Way. These natural light sources, as well as daily light/night cycles, play a fundamental role on behavioral patterns of marine and terrestrial organisms and the timing of ecological processes (Gaston, Bennie, Davies, & Hopkins, 2013; Gaston, Davies, Nedelec, & Holt, 2017; Longcore & Rich, 2004; Luarte et al., 2016). Artificial Light Pollution at Night (hereafter ALAN) is the alteration of natural light levels as the result of anthropogenic light sources (Cinzano, Falchi, & Elvidge, 2001; Duarte et al., 2019; Falchi et al., 2016).

Humans have been migrating to coastal regions and increasing population sizes on a global scale at a rate faster than the growth of the general population (Nicholls, 1995). This unequal distribution of population growth has led to the vulnerability of coastal habitats to increasing levels of light pollution (POL; Bird, Branch, & Miller, 2004; Garrett, Donald, & Gaston, 2019). It is most probable that POL is changing the structure and function of marine ecosystems in several key ways, which are in need of further study (Davies, Duffy, Bennie, & Gaston, 2014). ALAN is affecting roughly 22% of global coastlines (Davies et al., 2014; Underwood, Davies, & Queirós, 2017) and 35% (20% across their entire area) of marine‐protected areas (Davies et al., 2017). This further suggests that marine habitats and intertidal zones are vulnerable to the potential impacts of the disruption of natural day–night cycles that influence the behaviors of several marine species, including those that live in coral reefs (Duarte et al., 2019; Underwood et al., 2017).

Coral reefs are one of the most diverse and important marine ecosystems, providing homes to hundreds of thousands of species (Sebens, 1994), including almost a third of the world's marine fish species (Moberg & Folke, 1999). Coral reefs support more species per unit area than any other marine ecosystem, making them an important reservoir for biological diversity and complexity. The contribution of coral–microalgal (Symbiodiniaceae) mutualistic endosymbiosis to coral reefs rapid ecological success over history is profound (LaJeunesse et al., 2018; Muscatine et al., 2005). This relationship is closely tied to the ability of corals to deposit their calcium carbonate skeletons, thus allowing reef formation (Weis, Davy, Hoegh‐Guldberg, Rodriguez‐Lanetty, & Pringle, 2008). Many taxa on coral reefs are dependent on light–dark cycles, such as the expansion–contraction behavior in anthozoans to conserve nutrients (Levy, Mizrahi, Chadwick‐Furman, & Achituv, 2001; Sebens & DeRiemer, 1977), and diel vertical migrations of zooplankton and their planktivorous fish (Yahel, Yahel, Berman, Jaffe, & Genin, 2005). For example, Scleractinian corals and many other marine invertebrates synchronize reproduction by monthly patterns of lunar illumination (Bentley, Olive, & Last, 2001), which can be detected through high photosensitivity to low light intensity moonlight. ALAN has the potential to be strongly disruptive to such processes; nonetheless, the impact of POL on coral reefs remains largely unexplored, despite its potential to alter the coral physiology, symbiosis, and the reproductive timing (Kaniewska, Alon, Karako‐Lampert, Hoegh‐Guldberg, & Levy, 2015) on which corals depend for their reproduction and survival.

Over the past several decades, reefs throughout the world have been affected by anthropogenic climate change—as many as 75% of the world's coral reefs are threatened and as many as 95% may be in danger of being lost by mid‐century (Hoegh‐Guldberg, 2014). This could be attributed to mass bleaching events that are tied to global warming (Downs et al., 2012), but local stressors associated with overharvesting and coastal development (urban and agricultural) are also major contributors to this global decline (De'ath, Fabricius, Sweatman, & Puotinen, 2012). POL, such as ALAN, can be observed for fringing coral reefs in strongly urbanized locations, one example is the coastline in the Gulf of Eilat/Aqaba in the Red Sea (GOE/A). The GOE/A is heavily light polluted (with a geographical gradient from north to south) and the light reflected from the cities surrounding the reef (both Eilat in Israel and Aqaba in Jordan) can be seen from space (Tamir, Lerner, Haspel, Dubinsky, & Iluz, 2017). The reef in the GOE/A is of interest as it is the northernmost border for coral reefs and one of the most diverse reefs in the world.

Our study clearly shows that ALAN can impact coral physiology and photosynthesis. By using LEDs consisting of different light spectrums, we show reactive oxygen species (ROS) overproduction aligned with increasing levels of lipid damage, changes in the antioxidant capacity, decreasing electron transport rate (ETR), and alteration in chlorophyll and algae density in two key coral species, *Acropora eurystoma* and *Pocillopora damicornis*, from the GOE/A, Red Sea.

## 2 MATERIALS AND METHODS

### 2.1 Coral collection, maintenance, and sampling

Mature colonies of *A. eurystoma* and *P. damicornis* were collected by scuba diving at 4–5 m depths in the Gulf of Aqaba/Eilat Red Sea (28.6929°N, 34.7299°E) from artificial objects during 2017/2018. Experiments were conducted in 30 L aquaria partially submerged in an outdoor water table. Seawater was continuously pumped into each flow‐through aquarium, exchanging the water on average every 30 min. Corals were acclimated 21 days before experiments started. Submersible, Altman At‐301, pumps ensured adequate water mixing inside each aquarium. The water table area was covered with 70% shade net (30% transmitted Ambient light) in the winter and 60% shading (40% Ambient transmitted light) in the summer based on Levy et al. (2004). Intensity of the irradiance on the tables was measured using LI‐193 underwater Spherical Quantum Sensor. The irradiance on the table was equivalent to those experienced by corals at the collection depth, during the same seasonal period in which the experiment was conducted. Experimental temperatures and Ambient light inside the aquaria were monitored using data loggers (HOBO‐Onset data logger) throughout the entire experimental periods. In the first experiment (Exp 1, up to 120 days starting from April 2018), corals were divided into two groups and placed in 12 flow‐through aquarium systems. Each experimental group consisted of six coral colonies per species, the first group was natural light (*n* = 6), Ambient light cycle, and moon phase (AMB corals). The second group had artificial light contamination (ALAN corals, *n* = 6) from small White LED light strips 6,000–6,500 K (400–700 nm) with intensity of 1–1.5 µmol quanta m^−2^ s^−1^ (35–40 lux) that were turned on every day at sunset until sunrise by photocell. Light was measured using a LI‐COR underwater light meter quantuam sensor LI‐193, and spectrum measurements were made using an Ocean optics JAZ spectrometer (Figure S1). Light intensity in the aquariums was adjusted to mimic the same intensity of light that penetrates the water at 3‐5 m depth at the northern part of the Gulf of Aqaba/Eilat in the Red Sea. A black polygal was placed after sunset and was removed before sunrise between the aquarium systems to prevent light contamination among treatments. Corals were kept under experimental conditions for 4 months and sampling occurred in two time‐points, after 40 days (sample name T2) of exposure and at the end of the experiment (T6, 120 days). The second experiment (Exp 2, up to 20 days starting from November 2018) was conducted in a similar way as described above using three different LED lights—Blue (420–480 nm, 10,000 K), Yellow (580–620 nm, 2,000 K), and White (400–700 nm, 6,000–6,500 K), with intensity of 1–1.5 µmol quanta m^−2^ s^−1^ (35–40 lux) that were turned on every day at sunset until sunrise by photocell. Light was measured and adjusted using a LI‐COR underwater light meter quantuam sensor LI‐193; spectrum measurements were made using an Ocean optics JAZ spectrometer (Figure S1). During each experiment, fragments were sampled simultaneously from all treatments, the number of fragments that were sampled each time varied between five and six fragments per time point per light treatment. Following dark acclimation, fluorescence measurements (see Section 2.2) followed by physiological assays were conducted (see Section 2.4). In the first experiment (Exp 1), coral fragments were sampled after 40 and 120 days for total antioxidant capacity (TAC) and lipid peroxidation (LPO) measurements. In the second experiment (Exp 2), corals were sampled after 10 and 20 days of exposure, at different daylight hours (5 and 11 a.m.), for TAC, LPO, and ROS analysis.

### 2.2 Fluorescence measurements

Photosynthetic efficiencies were measured in corals with Imaging‐PAM (pulse amplitude modulation; Maxi‐PAM, Walz Gmbh). The resulting images were analyzed with the Imaging‐Win software program (v2.00 m; Walz Gmbh) and recorded for each of the branches. Rapid light curves (RLC) as ETR were measured with increasing illuminations of 120‐s intervals (0, 20, 55, 110, 185, 280, 335, 395, 460, 530, 610, 700 µmol quanta m^−2^ s^−1^) under an Ambient temperature. All fragments were dark‐adapted 20 min prior to the measurements. RLC‐driven parameter points were extracted using SigmaPlot Version 11 describing the shape of the curve: relative initial photosynthetic rate (*α*), relative maximum ETR through photosystem II (PSII) (rETRmax), compensation point (Ik; i.e., rETRmax divided by *α*; gives an indication of the irradiance at which absorbed quanta become dissipated through nonphotochemical quenching), and Im maximum saturating irradiance after (Ralph & Gademann, 2005).

### 2.3 Oxidative stress analyses

#### 2.3.1 Sample preparation

Small coral pieces (*N* = 5, per treatment) were cut (~0.3 cm^2^) and homogenized by ultrasound using 250–300 µl of the specific homogenization buffer for each analysis, as described below. Sonication was performed using an ultrasound water bath (Ultrasonic Cleaner; model Y‐008) filled with ice‐cold water. After sonication, the remaining skeleton was discarded, the holobiont, homogenate solution was centrifuged, and the intermediary phase was collected and immediately used for oxidative stress analysis. Total protein content of sample homogenates was determined according to (Bradford, 1976) using the Quick Start Bradford Protein Assay Kit (Bio‐Rad Laboratories Inc.).

#### 2.3.2 Reactive oxygen species

Reactive oxygen species quantification was performed using the fluorescent probe 2′,7′‐dichlorodihydrofluorescein (H_2_DCFDA; Invitrogen) according to de Aguiar et al. (2008), with some modifications. Briefly, samples were homogenized in a buffer containing Tris‐HCl 100 mmol/L (pH 7.75), ethylenediaminetetraacetic acid 2 mmol/L, and MgCl_2_ 5 mmol/L, and centrifuged for 20 min (20,000 *g*, 4°C). Sample protein content was adjusted to a final concentration of 0.5 mg/ml and 20 µl was added in a flat‐bottom black microplate containing the following medium: HEPES 30 mmol/L, KCl 200 mmol/L, and MgCl_2_ 1 mmol/L (pH 7.2). Finally, 10 µl of H_2_DCFDA 16 µmol/L was added and the fluorescence (excitation: 488 nm; emission: 525 nm) was measured every 5 min up to 50 min using a spectrofluorometer (Ultrospec 2100 pro). The results were expressed as fluorescence units per minute (F.U. × min).

#### 2.3.3 Total antioxidant capacity

Total antioxidant capacity measurement was determined using the “OxiSelect^TM^ TAC Assay Kit” (Cell Biolabs Inc.) according to the manufacturer's instructions. This assay measures the TAC of biomolecules via single electron transfer mechanism (Huang, Boxin, & Prior, 2005). Specifically, the commercial kit employed is based on the reduction of copper (II) to copper (I) by antioxidants, with marginal radical interference. Upon reduction, the copper (I) ion further reacts with a coupling chromogenic reagent with a maximum absorbance at 490 nm. The net absorbance values of antioxidants of coral samples were compared with a known uric acid standard curve, with absorbance values being proportional to the sample's total reductive capacity. Absorbance readings were performed in a 96‐well flat‐bottom transparent microplate using spectrofluorometer (Ultrospec 2100 pro). Data were normalized considering the total protein content in the sample homogenates in each well and expressed as µmol L^−1^ copper reducing equivalents mg protein^−1^.

#### 2.3.4 Lipid damage

Lipid damage (as LPO) quantification was performed according to the method described by Oakes and Van Der Kraak (2003). Samples were homogenized in KCl (1.15%) solution containing 35 µmol/L butylated hydroxytoluene and centrifuged for 10 min (10,000 *g*, 4°C). This method is based on the 2‐thiobarbituric acid reactive substances, and quantifies the peroxidative damage to lipids through the reaction between malondialdehyde (MDA), a byproduct of LPO, and thiobarbituric acid. The reaction, at high temperature and acidity, generates a chromogen that is measured by spectrofluorometry (excitation: 515 nm; emission: 553 nm). Measurements were performed in a 96‐well flat‐bottom black microplate using a spectrofluorometer (Ultrospec 2100 pro). Data were normalized considering the total protein content in the sample homogenates in each well and expressed as nmol MDA/mg protein.

### 2.4 Physiological assays

#### 2.4.1 Separation of coral tissue and symbiotic algae

Coral fragments were taken out of the −80°C freezer and slowly defrosted in an ice bucket. Coral tissue was separated from the skeleton using an airbrush connected to a compressed air diving tank and a 5 ml beaker of 0.2 μm filtered sea water (FSW; Johannes & Wiebe, 1970). FSW was filtered through a 0.2 μm pore size with 25 mm diameter polycarbonate filter using a vacuum pump (Rocker 300; Rocker Scientific Co. Ltd). The FSW containing the coral tissue extracts was collected into a 50 ml Falcon tube and the total volume was measured. The coral skeleton was dried for 24 hr (at a temperature of 60°C) and was later used for surface area measurement (see Section 2.4.4). The FSW containing the tissue extracts was homogenized by an electrical homogenizer (Diax 100 homogenizer, Heidolph Instruments GmbH and Co. KG) for 20 s and 100 μl of the homogenate was collected and stored in a 0.5 ml Eppendorf tube for total protein analysis (see Section 2.4.5). Half of the total amount was then separated (spare) and stored in a −80°C freezer. The rest of the homogenate in each 50 ml Falcon was centrifuged (Sigma 4k15; Sigma Laboratory Centrifuges) for 5 min at 2000 *g* at 4°C. Hundred microliters of the supernatant was transfered to a 0.5 ml Eppendorf tube for host protein analysis (see Section 2.4.5). The supernatant was removed and the pellet containing the algae was resuspended in 5 ml of FSW. The sample was then homogenized for 20 s and centrifuged for an additional 5 min at 2000 *g* at 4°C. The supernatant was removed while the pellet was resuspended in 1 ml of FSW and transferred into a 1.5 ml Eppendorf tube. The sample was homogenized and centrifuged, the supernatant was removed and the pellet was resuspended in 1 ml of FSW and homogenized again. Hundred microliters of processed sample was removed for algae cell count. The rest of the sample (900 μl) was vortexed (Biosan Bio Vortex V1) and centrifuged, and the supernatant was removed thoroughly. One milliliter of 90% acetone was added to each chlorophyll sample. The tube was vortexed and incubated for 15 hr at 4°C in the dark.

#### 2.4.2 Algal chlorophyll

To measure the chlorophyll concentration in the symbiotic algae, chlorophyll samples were vortexed and centrifuged for 5 min at 2000 *g* at 4°C and placed on ice in the dark. The optical density (OD) of each sample supernatant was measured by a Multiskan Spectrum Plate Reader (Multiskan Spectrum; Thermo Scientific) at three different OD: 630, 663, 750 nm. Blank measurements (90% Acetone) were subtracted from the obtained results. If the readings were above OD 1.00, the samples were diluted. The concentration of chlorophyll was calculated according to the spectrographic method (Jeffrey & Humphrey, 1975). The chlorophyll *a* concentration (μg/ml) was calculated according to the appropriate equation (see below). The results were normalized to algae count (see Section 2.4.3) to determine the amount of chlorophyll per algal cell (μg/cell) and to surface area (see Section 2.4.4) to determine the amount of chlorophyll to area (μg/cm^2^).

#### 2.4.3 Algal count

Symbiotic algae were counted using digital photographs of a microscope image. For this purpose, samples were unfrozen on ice and vortexed before counting to avoid both settling and clumping of the cells. In cases of high algae density, the samples were diluted to obtain more reliable counts. From each fragment, a sample of 100 μl was taken and placed on a hemacytometer (Fitt, Spero, Halas, White, & Porter, 1993; Muscatine, Falkowski, Dubinsky, Cook, & McCloskey, 1989). A Marienfeld 0.0025 mm^2^ hemacytometer with two counting areas was used. Each counting area with four fields composed of 16 squares each. Four fields from each area were chosen (all the corner squares) and photos were taken through a microscope (Nikon Eclipse TE 2000‐E; Nikon) under ×100 magnification, with four squares present in each picture (eight square fields, 32 pictures for each sample). The algal cells in the photos were counted manually using ImageJ© program (Cell Counter application) and the count of each field was summed together. The sum of each of the eight fields was averaged, and the value was multiplied by 10,000 (each field size is 0.1 cm × 0.1 cm × 0.01 cm depth) to calculate the number of symbiotic algae present in each sample.

#### 2.4.4 Surface area

Surface area was determined for *A. eurystoma* and *P. damicornis* by the paraffin wax method of Stimson and Kinzie (1991). Surface area was measured by dipping each fragment in hot wax (65°C) for 3 s. It was then cooled to room temperature to allow the wax to solidify. The net weight (weight after dipping weight before) was multiplied by a coefficient number, which was obtained by using the same coating method on cylinders with known surface area and calibrating against a regression coefficient (*R* = .978, *n* = 34).

#### 2.4.5 Protein concentration analysis

Protein (total and host) concentrations were measured according to Bradford (1976) using the Quick Start Bradford Protein Assay Kit (Bio‐Rad Laboratories Inc.). Each total protein sample was sonicated (Branson Sonifier B‐12) for 10 s on ice to break the algae cells. A blank of FSW 0.4 μm was created and used as a reference for the samples. A standard curve was created using six increasing concentrations of Quick Start bovine serum albumin standard set (cat #500‐0207; Bio‐Rad Laboratories). Concentrations of 0.125, 0.25, 0.5, 0.75, 1, and 1.5 mg/ml were used. A blank of double distilled water (DDW) was created and used as a reference for the standard curve. Triplicates of 5 μl from each sample were added to a 96‐well plate. Two hundred microliters of Bradford dye reagent was added to the samples in a 96‐well plate followed by a 30‐min dark incubation. The plate was loaded into a Multiskan Spectrum Plate Reader (Multiskan Spectrum; Thermo Scientific). The OD of each well was read at 595 nm. The OD of the standard curve was plotted (after the triplicates were averaged and the DDW blank was subtracted) and protein concentration equation was derived from the linear curve. Triplicates of each sample were averaged, FSW blank was subtracted, and protein concentration was calculated. The protein concentration of each sample was then multiplied by the total sample volume to get the total amount of protein. Total and host protein were normalized to surface area. The obtained ratios represent changes in the holobiont and coral biomass, respectively.

### 2.5 Statistical analysis

The statistical analysis was conducted using the R statistical environment (Jombart, 2008). Nonlinear relationship between irradiance and ETRs under different treatments (or time‐points) was modeled with a linear mixed‐effects regression. A third‐degree polynomial term for irradiance was included in the model. Treatments (or time‐points) were treated as a fixed factor and coral identification number was treated as a random effect. Treatments (or time‐points) were compared in terms of differences between intercepts (contrasts). Confidence intervals for fixed effects and contrasts were bootstrapped. *p*‐Values, the fixed effect estimates, were obtained by *t* tests using Satterthwaite approximations to degrees of freedom and corrected for multiple comparisons using the FDR procedure. Homoscedasticity and normality of residuals were inspected visually. Effects of time and POL on oxidative stress parameters (ROS, TAC, and LPO) were evaluated using two‐way ANOVA. If indicated, ANOVA was followed by post hoc Student–Newman–Keuls (SNK) test. Homogeneity of variances and data normality were checked prior to the analysis using Levene and Shapiro–Wilk tests, respectively. Data were log‐transformed to meet ANOVA assumptions when necessary. In all cases, the significance level adopted was 95% (*α* = 0.05). Results were expressed as mean ± *SE*.

## 3 RESULTS

### 3.1 Electron transport rate

**Figure 1:**
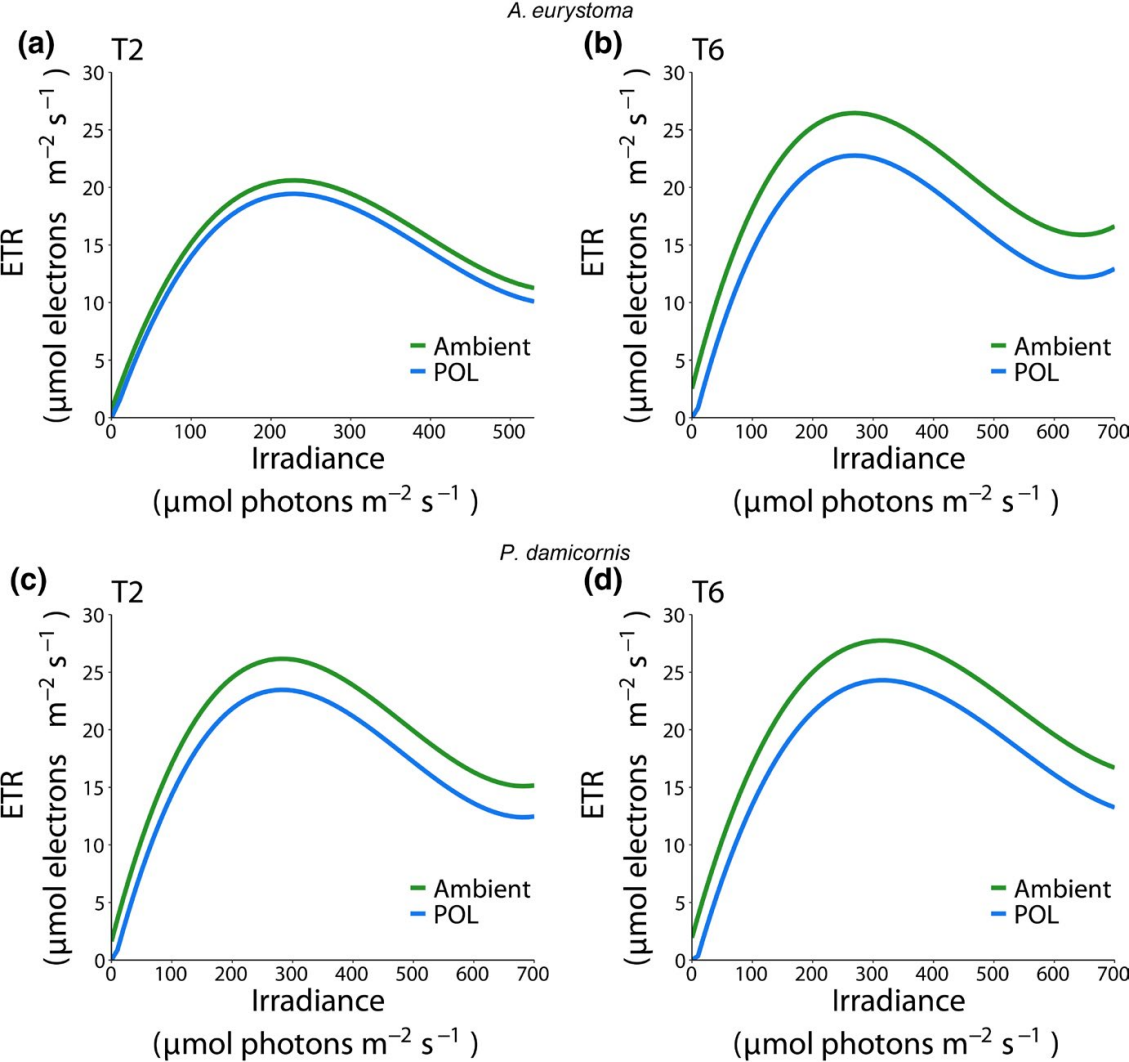
Relationship between irradiance and electron transport rate (ETR) in the corals *Acropora eurystoma* (a, b) and *Pocillopora damicornis* (c, d) under different light conditions (Ambient and light pollution [POL]) after different periods of exposure (Exp 1:40 [T2] and 120 [T6] days). Predicted fit obtained from third‐degree polynomial linear mixed model

**Figure 2:**
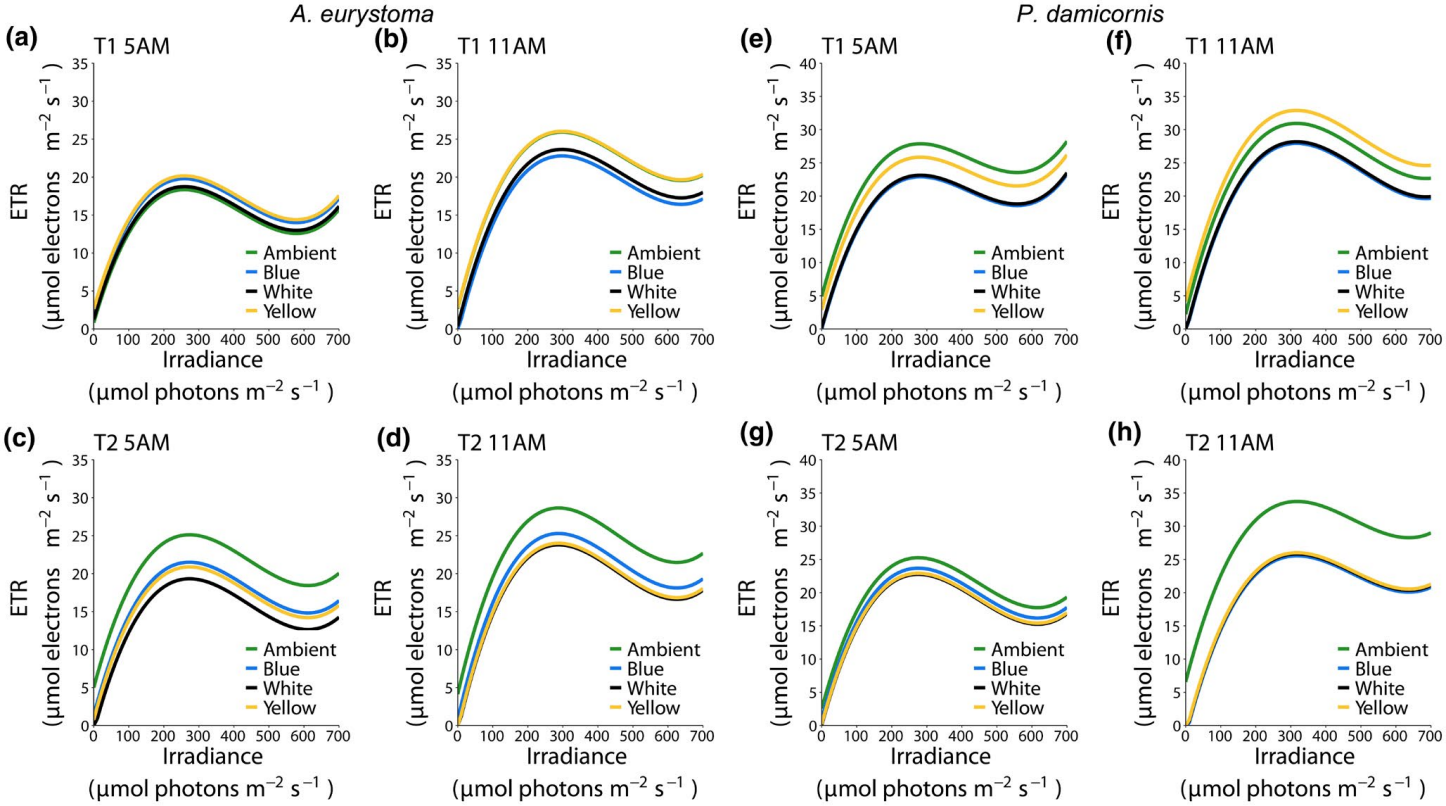
Relationship between irradiance and electron transport rate (ETR) in the corals *Acropora eurystoma* (a–d) and *Pocillopora damicornis* (e–h) exposed to different monochromatic light conditions (Ambient, Blue, White, and Yellow lights) at different daylight hours (5 and 11 a.m.) and times of exposure (Exp 2:10 [T1] and 20 [T2] days]. Predicted fit obtained from third‐degree polynomial linear mixed model. Mean ETR ± *SEM* for each irradiance levels are presented in Figure S3

Photochemical measurements of ETR significantly decreased when corals were exposed to artificial light in both experiments. In general, ETR increases as irradiance level increases, until a maximum level is reached (around 300 µmol photons m^−2^ s^−1^). After this point, ETR levels decrease in a more moderate manner. We thus modeled this nonlinear relationship with linear mixed models to measure ETR performance, as a function of increased irradiance levels. Next, we analyzed the impact of exposure to POL (Figure 1, see also raw data Figure S2) on symbionts chlorophyll fluorescence as a measurement of ETR under POL in comparison to control (Ambient). The results show a reduction in both *A. eurystoma* and *P. damicornis* at all time‐points (*p* < .05 for all comparisons; Table 1). Specifically, in experiment 1, the difference increased at time‐point 6 (difference between curves of 3.69 and 3.45 [μmol electrons m^−2^ s^−1^] in ETR in *A. eurystoma* and *P. damicornis*, respectively). The summarized values of the RLC measured parameters (Table S1) only on the average samples showing differences in the rETR, Im, *α*, and Ik with a decreasing performance in the POL corals of both species. We then tested whether subspecies Blue, White, and Yellow have a different effect on ETR, compared against the Ambient at different time‐points in *A. eurystoma* (Figure 2a–d, see also raw data Figure S3), experiment 2. At time‐point 1 (T1), a small, but significant decrease (compared to Ambient) was observed only at 11 a.m. in blue and white (difference of 3.14 and 2.29 [μmol electrons m^−2^ s^−1^], *p* = .003 and .0249, respectively), but no difference for Yellow. At time‐point 2 (T2; both 5 and 11 a.m.), all subspecies had significantly lower ETR levels (Table 2). We repeated the same analysis in *P. damicornis* (Figure 2e–h, see also raw data Figure S3). In contrast with *A. eurystoma*, a greater decrease was already observed at 5 a.m. (T1) in Blue and White (difference of 4.98 and 4.74 [μmol electrons m^−2^ s^−1^], respectively, *p* = .003 for both comparisons), but again not in Yellow (Table 3). At T2, at 5 a.m., Blue was not any different than the Ambient, but ETR levels in Yellow and White were significantly lower (difference of 2.33 and 2.49 [μmol electrons m^−2^s^−1^], *p* = .0315 and *p* = .0299, respectively). At 11 a.m., higher ETR levels were observed in Ambient, leading to greater difference with blue, White, and Yellow (difference of 8.18, 7.9, 7.72 [μmol electrons m^−2^ s^−1^], respectively, *p* < .001 for all comparisons). The photochemical parameters of the RLC for both corals are presented in Tables S2 and S3, summarized on the average samples, showing as lower performances mainly under the Blue and White light treatments in both sampling time‐points T1 and T2 and at the different hours measured.

**Table 1:** Comparison between Ambient and light pollution (POL) curves obtained from linear mixed models, represented as estimated intercept difference with bootstrapped confidence interval and *p* value of statistical testing for significant difference from 0

	*Acropora eurystoma* T2	*Acropora eurystoma* T6	*Pocillopora damicornis* T2	*Pocillopora damicornis* T6
POL–Ambient	−1.1653 (−2.2806, −0.021) *p* = .048*	−3.6915 (−5.1362, −2.3974) *p* < .001***	−2.7002 (−3.7624, −1.7286) *p* < .001***	−3.4509 (−5.4193, −1.2142) *p* = .003**

Note

This table is related to Figure 1.

* *p* = 0.05;

** *p* = 0.01;

*** *p* = 0.001.

**Table 2:** Comparison between fitted curves in *Acropora eurystoma* obtained from linear mixed models, represented as estimated intercept difference with a bootstrapped confidence interval and *p* value of statistical testing for significant difference from 0

	T1	T1	T2	T2
	5 a.m.	11 a.m.	5 a.m.	11 a.m.
Blue–Ambient	1.4385 (−0.4122, 3.2892) *p* = .1355	−3.1411 (−5.5957, −0.6864) *p* = .0030**	−3.6147 (−6.1962, −1.0332) *p* = .0007***	−3.3624 (−6.7266, 0.0018) *p* = .0204*
White–Ambient	0.4042 (−1.4941, 2.3025) *p* = .6193	−2.2858 (−4.7397, 0.1681) *p* = .0249*	−5.7982 (−8.6449, −2.9515) *p* = .0000***	−4.8522 (−8.2854, −1.4190) *p* = .0012**
Yellow–Ambient	1.8127 (−0.0856, 3.7111) *p* = .0850	0.0983 (−2.3546, 2.5513) *p* = .9179	−4.2339 (−7.0812, −1.3866) *p* = .0004***	−4.6429 (−8.0087, −1.2770) *p* = .0012**
White–Blue	−1.0343 (−2.9693, 0.9008) *p* = .2547	0.8553 (−1.6010, 3.3115) *p* = .4448	−2.1835 (−4.6811, 0.3140) *p* = .0375*	−1.4898 (−4.7744, 1.7947) *p* = .3655
Yellow–Blue	0.3743 (−1.5608, 2.3093) *p* = .6193	3.2394 (0.7840, 5.6949) *p* = .0030**	−0.6192 (−3.1174, 1.8790) *p* = .5251	−1.2805 (−4.4946, 1.9337) *p* = .3670
Yellow–White	1.4085 (−0.5722, 3.3892) *p* = .1355	2.3842 (−0.0705, 4.8388) *p* = .0249*	1.5643 (−1.2071, 4.3357) *p* = .1774	0.2094 (−3.0769, 3.4957) *p* = .8699

Note

This table is related to Figure 2a–d.

* *p* = 0.05;

** *p* = 0.01;

*** *p* = 0.001.

**Table 3:** Comparison between fitted curves in *Pocillopora damicornis* obtained from linear mixed models, represented as estimated intercept difference with bootstrapped confidence interval and *p* value of statistical testing for significant difference from 0

	T1	T1	T2	T2
	5 a.m.	11 a.m.	5 a.m.	11 a.m.
Blue–Ambient	−4.9758 (−8.1897, −1.7619) *p* = .0003***	−3.0150 (−6.9806, 0.9507) *p* = .0985	−1.5707 (−3.8521, 0.7106) *p* = .1536	−8.1838 (−11.6508, −4.7167) *p* < .001***
White–Ambient	−4.7371 (−7.8650, −1.6093) *p* = .0003***	−2.7649 (−6.6227, 1.0929) *p* = .0985	−2.4930 (−4.7750, −0.2111) *p* = .0299*	−7.9000 (−11.3852, −4.4148) *p* < .001***
Yellow–Ambient	−2.0260 (−5.1641, 1.1121) *p* = .1167	1.9472 (−1.8216, 5.7160) *p* = .2215	−2.3324 (−4.6745, 0.0098) *p* = .0315*	−7.7218 (−11.0268, −4.4169) *p* < .001***
White–Blue	0.2386 (−2.9646, 3.4419) *p* = .8482	0.2501 (−3.7217, 4.2219) *p* = .8715	−0.9223 (−3.1425, 1.2979) *p* = .4285	0.2838 (−3.2095, 3.7770) *p* = .8908
Yellow–Blue	2.9498 (−0.2623, 6.1619) *p* = .0366*	4.9621 (1.0767, 8.8475) *p* = .0040**	−0.7617 (−3.0438, 1.5205) *p* = .4692	0.4620 (−2.8512, 3.7751) *p* = .8908
Yellow–White	2.7112 (−0.4148, 5.8371) *p* = .0388*	4.7120 (0.9369, 8.4872) *p* = .0040**	0.1607 (−2.1221, 2.4435) *p* = .8565	0.1782 (−3.1543, 3.5106) *p* = .8908

Note

This table is related to Figure 2e–h.

* *p* = 0.05;

** *p* = 0.01;

*** *p* = 0.001.

### 3.2 Oxidative stress analysis

#### 3.2.1 Experiment 1

**Figure 3:**
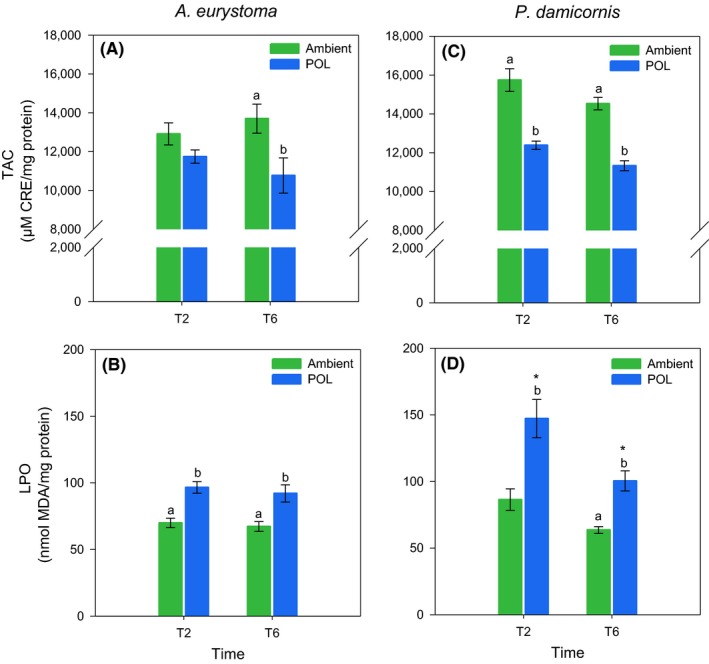
Total antioxidant capacity (TAC) and lipid peroxidation (LPO) in the coral holobionts *Acropora eurystoma* (A, B) and *Pocillopora damicornis* (C, D) exposed to different light conditions (Ambient and light pollution [POL]) at different times of exposure (Exp 1: 40 [T2] and 120 [T6] days). Data are expressed as mean ± *SEM* (*n* = 5). Different lowercase letters indicate significantly different mean values (*p* < .05) between treatments in the same time of exposure. Asterisks (*) indicate significantly different mean values (*p* < .05) between the same treatments in different times of exposure

Significant differences were indicated for the factor treatment regarding *A. eurystoma* mean TAC (ANOVA, *p* = .001). No changes in *A. eurystoma* TAC were observed between treatments at T2 (SNK, *p* = .15). In turn, a significant decrease in TAC was observed at T6 in corals under the POL treatment (SNK, *p* < .001; Figure 3A). Concerning mean LPO, significant differences were indicated between treatments (ANOVA, *p* = .001). Increased LPO was observed in corals under POL treatment at both times of exposure (SNK, *p* ≤ .01; Figure 3B). Significant differences between treatments were indicated for *P. damicornis* mean TAC at T2 (40 days) and T6 (120 days; ANOVA, *p* ≤ .001), with lower values observed for the POL treatment (SNK, *p* < .001; Figure 3C). Significant differences were also indicated for LPO with respect to Time (ANOVA, *p* = .006) and Treatment (ANOVA, *p* < .001) factors. In alignment with corals decreased TAC, an increase in LPO was observed for the polluted treatment at both times of exposure (SNK, *p* ≤ .006; Figure 3D). However, corals' LPO mean values decreased over time under artificial light treatment (SNK, *p* = .02; Figure 3D).

#### 3.2.2 Experiment 2

**Figure 4:**
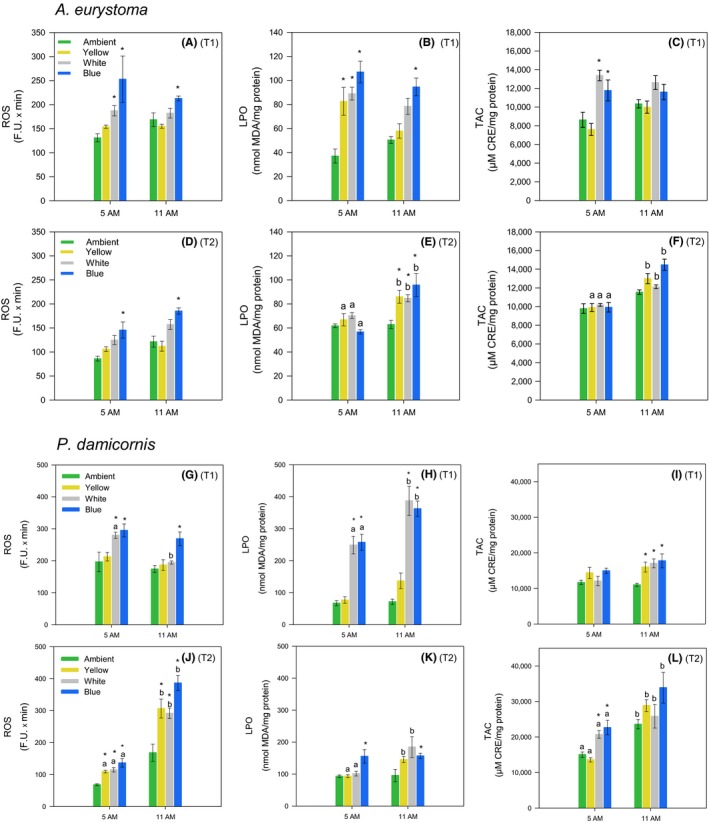
Reactive oxygen species (ROS), lipid peroxidation (LPO), and total antioxidant capacity (TAC) in the coral holobionts *Acropora eurystoma* (A–F) and *Pocillopora damicornis* (G–L) exposed to different monochromatic light conditions (Ambient, Yellow, White, and Blue lights) during different daylight hours (5 and 11 a.m.) and times of exposure: Exp 2, T1 (10 days), (A–C and G–I) and Exp 2, T2 (20 days), (D–F and J–L). Data are expressed as mean ± *SE* (*n* = 5). Asterisks (*) indicate significantly different mean values (*p* < .05) between light treatments and the Ambient condition at same daylight hour. Different lowercase letters indicate significantly different mean values (*p* < .05) between the same light treatments at different daylight hours

Significant differences among light treatments were indicated for all oxidative stress parameters measured in the coral holobiont *A. erystoma* after 10 days (T1) of exposure (ANOVA, *p* < .001). Overproduction of ROS was observed in corals at 5 a.m. under White and Blue light treatments with respect to Ambient condition (SNK, *p* ≤ .01); however, at 11 a.m., only corals under the Blue light treatment presented significant higher ROS production (SNK, *p* < .04; Figure 4A). Increased LPO was observed at 5 a.m. in all light treatments compared to the Ambient condition (SNK, *p* ≤ .01; Figure 4B). At 11 a.m., corals under Blue light treatments still presented higher LPO (SNK, *p* ≤ .01; Figure 4B). Concerning TAC, increased values were observed at 5 a.m. for White and Blue light treatments with respect to the Ambient condition (SNK, *p* ≤ .01; Figure4C). After 20 days (T2), significant differences among light treatments were indicated for *A. erystoma* mean ROS values (ANOVA, *p* < .001). Overproduction of ROS was observed in corals under Blue light treatment at both daylight hours (SNK, *p* ≤ .02; Figure 4D). Significant differences among treatments, as well as a time effect, were also indicated for *A. erystoma* mean LPO (ANOVA, *p* < .02). Corals' LPO increased over time in all light treatments (SNK, *p* ≤ .01; Figure 4E), with higher LPO values being observed with respect to Ambient condition at 11 a.m. (SNK, *p* < .05; Figure 4E). Concerning TAC, only a time effect was indicated (ANOVA, *p* < .001), with higher TAC observed in corals under all light treatments at 11 a.m. (SNK, *p* ≤ .04; Figure 4F). Significant differences among treatments, as well as a time effect, were indicated for all oxidative stress parameters measured in the coral holobiont *P. damicornis* after 10 days (T1) of exposure (ANOVA, factor Time, *p* ≤ .04, factor Treatment, *p* ≤ .02). Overproduction of ROS was observed at 5 a.m. in corals under the White and Blue light treatments with respect to the Ambient condition (SNK, *p* < .05; Figure 4G). At 11 a.m., only corals in the Blue light treatment presented higher ROS levels (SNK, *p* < .06), while the ones under the White light treatment showed a decrease in ROS over daylight time (SNK, *p* ≤ .01; Figure 4G). Increased LPO was observed for the White and Blue light treatments at both daylight times (SNK, *p* < .001), with a significant increase over time (SNK, *p* ≤ .03; Figure 4H). At 11 a.m., increased values of the corals' TAC were observed in all light treatments with respect to the Ambient condition (SNK, *p* ≤ .02; Figure 4I). After 20 days (T2), significant differences were also indicated among treatments as well as a time effect for all oxidative stress parameters measured in *P. damicornis* (ANOVA, factor Time, *p* ≤ .01, factor Treatment, *p* ≤ .01). The corals' ROS generation was higher in all light treatments compared to the Ambient condition at 5 a.m. (SNK, *p* ≤ .007) and 11 a.m. (SNK, *p* ≤ .003). Also, an increase in ROS generation over daylight time was observed in corals under all conditions (SNK, *p* < .001; Figure 4J). Regarding LPO, corals in the Blue light treatment presented significantly higher levels compared to the ones at the Ambient condition, at both daylight times (SNK, *p* ≤ .03; Figure 4K). In turn, the corals in the Yellow and White light treatments showed increased LPO over time (SNK, *p* ≤ .02), with significantly higher levels compared to the Ambient condition at 11 a.m. (SNK, *p* < .001; Figure 4K). Corals' TAC only showed higher values for the White and Blue light treatments with respect to the Ambient condition, at 5 a.m. (SNK, *p* ≤ .06; Figure 4L). In turn, aligned with increased ROS generation, all conditions presented a significant increase in TAC levels over daylight time (SNK, *p* ≤ .02; Figure 4L).

### 3.3 Physiology measurements

**Figure 5:**
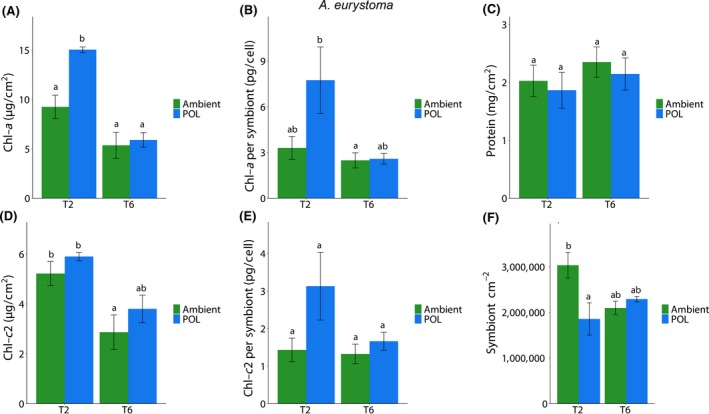
Effect of different light conditions (Ambient and light pollution [POL]) at different times of exposure (40 [T2] and 120 [T6] days) in the coral *Acropora eurystoma* in several parameters. Data are expressed as mean ± *SE* (*n* = 4–6). Different lowercase letters indicate significantly different mean values (*p* < .05). (A) Chlorophyll *a* (Chl‐*a*) per coral sample, (B) Chl‐*a* per algae cell, (C) total protein per coral sample, (D) Chlorophyll *c*2 (Chl‐*c*2) per coral sample, (E) Chl‐*c*2 per algae cell, (F) algae density per coral sample

**Figure 6:**
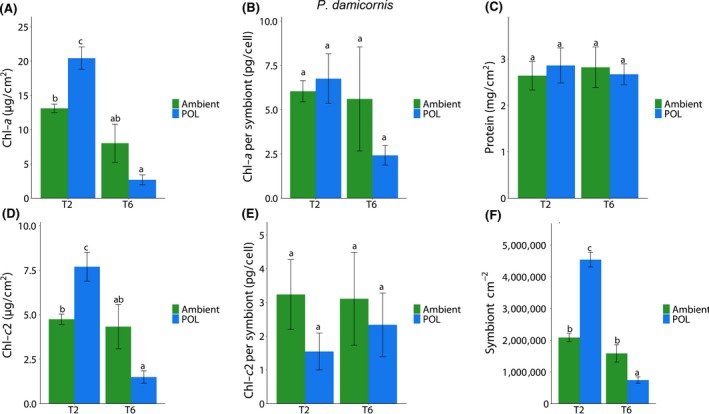
Effect of different light conditions (Ambient and light pollution [POL]) at different times of exposure (40 [T2] and 120 [T6] days) in the coral *Pocillopora damicornis* in several parameters. Data are expressed as mean ± *SE* (*n* = 4–6). Different lowercase letters indicate significantly different mean values (*p* < .05). (A) Chlorophyll *a* (Chl‐*a*) per coral sample, (B) Chl‐*a* per algae cell, (C) total protein per coral sample, (D) Chlorophyll c2 (Chl‐*c*2) per coral sample, (E) Chl‐*c*2 per algae cell, (F) algae density per coral sample

Several indexes regarding coral physiology were examined at the different sampling points of the experiment to evaluate the effect of ALAN on corals health. In the first experiment, physiology assay showed in *A. eurystoma* (Figure 5) a significant difference in chlorophyll parameters during T2 showing higher concentrations in the POL samples, including total Chl‐*a* concentration normalized to symbiont cell (pg total chl/cell) and total Chl‐*a* concentration normalized to surface area (µg total chl‐*a*/cm^2^). Symbiont cell concentration normalized to surface area showed a significant decrease during T2 phase in the POL samples. No differences were witnessed as for total protein concentration (mg total protein/cm^2^) nor Chl‐*c*2 concentration normalized to symbiont cell (pg total chl/cell). *P. damicornis* samples during the long‐term experiment (Exp 1) did not show any significant differences in total protein concentration, and in Chl‐*a* and *c* concentrations normalized to symbiont cell (pg total chl/cell). However, higher concentration normalized to surface area in Chl‐*a* and Chl‐*c*2 (µg total chl/cm^2^) was measured during T2 sampling point including higher symbiont density in POL samples. A significant decrease was recorded in all three parameters during T6 under the POL samples (Figure 6). In the short‐term experiment under Blue, White, Yellow LEDs, several parameters were significantly changed in *A. eurystoma* samples in comparison to the Ambient (Figure S4). Chlorophyll‐*a* concentration normalized to symbiont cell (pg/cell) under the Ambient treatment was significantly higher relative to the rest of the monochromatic lights, while symbiont cell concentration normalized to surface area (cm^–2^) showed a significantly lower density compared to the other light treatments. As for the Chl‐*a* concentration normalized to surface area (µg/cm^2^), there was a significant increase under the Blue wavelength. No differences for protein concentration (mg/cm^2)^ as well as for Chl‐*c*2 normalized to surface (µg/cm^2^) and Chl *c*2 per symbiont (pg/cell) were observed. In *P. damicornis* during the short‐term experiment, a significant decrease (Figure S5) in symbiont cell concentration normalized to surface area was recorded in Blue and White treatments compared to the Ambient and Yellow treatment. Chl‐*c*2 per symbiont (pg/cell) was significantly increased in the White and Blue. Chlorophyll‐*a* concentration normalized to symbiont cell (pg/cell) and Chl‐*a* concentration normalized to surface area (µg/cm^2^) did not show any significant changes in all treatments as well as for the protein concentration (mg/cm^2^; Figure S5).

## 4 DISCUSSION

### 4.1 Electron transport rate

Shift in electron flow in corals is shown to be primarily driven by light (Hoogenboom, Campbell, Beraud, DeZeeuw, & Ferrier‐Pagès, 2012). Despite photosystem I (PSI) ETR was not measured in the present study, the decreasing levels of PSII ETR in corals under POL conditions, herein observed suggests a transition from linear electron flow through PSII and PSI toward PSI dominated. In fact, cyclic electron flow, which involves only PSI (Allen, 2003), has been implicated in photoprotection by generating a proton gradient able to dissipate excess excitation energy from PSII (Heber & Walker, 1992; Hoogenboom et al., 2012; Johnson, 2004). Considering that light stress destabilization of the photosynthetic electron transport chain may result in increased ROS production (Richter, Rühle, & Wild, 1990), the observed decreasing PSII ETR including RLC parameters (Tables S1–S3) may be a physiological response to counteract the overproduction of ROS levels induced by the exposure to POL. Indeed, decreasing PSII ETR showed to be aligned, for both species, including decreasing TAC (which suggests increased ROS production) while increasing LPO in Exp 1, and an increasing of ROS and LPO levels in Exp 2. In experiment 2, these responses were more pronounced in corals under Blue and White light wavelengths, which were shown to be more harmful to corals (see Figure 4).

### 4.2 Oxidative stress responses to POL

Oxidative stress is a physiological condition where there is an imbalance between ROS and antioxidants within an organism. Excessive ROS accumulation leads to cellular injuries, such as damage to the genetic material, proteins, and lipid membranes (Lesser, 2006). Results from Exp 1 bring evidence that both coral species tested are facing an oxidative stress condition due to POL. This statement is based on increased levels of oxidative damage (here shown as LPO) aligned with lower TAC in corals exposed to ALAN. TAC decreased values indicate that more antioxidants are being consumed to counteract an overproduction of ROS. Augmented LPO levels reinforce this, which suggests that the coral antioxidant apparatus is not able to cope with excessive ROS formation, with a consequential increase in oxidative damage. Exp 2 shows that the most deleterious wavelengths affecting corals are those under the Blue light treatment. This is manifested by increasing levels of ROS and LPO observed for both species in all daylight measurements (5 and 11 a.m.) and lengths of exposure (10 and 20 days; Figure 4). The White light treatment also led to deleterious effects on corals, which showed increased ROS production and oxidative damage after 10 and 20 days of exposure. More specifically, *P. damicornis* is apparently more sensitive to the White light wavelengths, since ROS generation and LPO were more often observed for this species at both times of exposure. Regarding the Yellow light treatment, it seemed to be less aggressive to the oxidative status of corals. However, it is important to note that an increase in oxidative damage to lipids was observed after 20 days of exposure for both species at 11 a.m., even though TAC was observed to significantly increase over daylight time. In contrast to Exp 1, a trend of increase in TAC was observed in corals under all light treatments in Exp 2, suggesting that the antioxidant apparatus is being induced by excessive ROS production. These opposite TAC responses may be related to the different experimental lengths of exposure and the monochromatic wavelengths used for the POL treatments. In either case, significant variations in the endogenous levels of antioxidants under POL treatments (compared to the Ambient condition) can be interpreted as a stress‐induced ROS modulation to maintain cell homeostasis (Gardner et al., 2017; Huang et al., 2005). In this context, it is possible to infer that decreased levels of endogenous antioxidants could be expected after a period of oxidative imbalance, or a more intense stress situation experienced by organisms. However, varying responses of TAC among species, light treatments, daylight hours, and lengths of exposure observed indicate that a more complex antioxidant defense mechanism is involved in coral physiological response to POL. Antioxidant defenses are composed by enzymatic and nonenzymatic antioxidant systems that operate in both host and endosymbionts simultaneously, to modulate stress‐induced ROS and maintain cell homeostasis (Gardner et al., 2017). Considering that only the nonenzymatic antioxidant system was measured in the present study, the enzymatic system may act significantly in the antioxidant mechanism against ALAN. In fact, antioxidant enzymes such as super oxide dismutase and catalase have shown increased activity in corals exposed to increasing light levels (Higuchi, Agostini, Casareto, Suzuki, & Yuyama, 2016; Levy, Achituv, Yacobi, Stambler, & Dubinsky, 2006; Richier, Rodriguez‐Lanetty, Schnitzler, & Weis, 2008). Additionally, wavelength dependence of this two free radical scavenger enzyme activity previously revealed an increase in activity in the Blue light range (440–480 nm) compared to the Red (640–680 nm) in the full visible light (400–700 nm) range (Levy et al., 2006).

### 4.3 Physiological responses

Both species presented an increase in photosynthetic pigments after 40 days of exposure to POL in Exp 1; however, *A. eurystoma* presented an increase of Chl‐*a* (per symbiont) paralleled by a decrease in symbiont density, while *P. damicornis* showed an increase in Chl‐*a* and *c* (per coral surface area) aligned with an increase in symbiont density (Figures 5 and 6). These responses were followed by increases in oxidative damage (LPO); however, LPO was ~35% higher in *P. damicornis* compared to *A. eurystoma.* Excess algal symbionts may increase the susceptibility of corals to bleaching by generating more ROS on a per‐cell basis (Cunning & Baker, 2013; Nesa & Hidaka, 2009). Thus, the higher symbiont density presented by *P. damicornis* due to POL could have led to augmented ROS levels in the holobiont, which led to higher levels of LPO. Also, this statement is reinforced by the fact that after 120 days of exposure, *P. damicornis* presented a significant decrease in symbiont density exposed to the ALAN condition and LPO levels also decreased over time reaching similar levels shown by *A. eurystoma.* Changes in photosynthetic pigments were less prominent in Exp 2 and coral species responded differently to light treatments regarding symbiont density, with evidence that *P. damicornis* is more sensitive to light at night. *P. damicornis* showed a decrease of symbionts in the White and Blue light treatments, which is aligned with a more severe oxidative stress condition observed in these treatments, coupled by higher ROS and LPO levels, throughout the experiment (Figures S4 and S5). In contrast, all wavelengths tested caused an increase in *A. eurystoma* symbiont density and an increase in LPO levels, which is in accordance with observations from Exp 1, that indicated that a higher symbiont density resulting from ALAN may increase ROS content in the holobiont. It is worth noting that overall levels of ROS and LPO were higher in *P. damicornis* compared to *A. eurystoma* when exposed to POL conditions during Exp 2 (Figure 4). This observation can elucidate, at least in part, the higher sensitivity of *P. damicornis* host to LPO compared to *A. erystoma*, which we do not think it is directly related to the coral host symbiotic algae since both species are associated with Cladocpium (clade C; Karako‐Lampert, Katcoff, Achituv, Dubinsky, & Stambler, 2004).

Today, there is still a significant knowledge gap regarding the diversity of taxa and habitats impacted by ALAN or ecological POL (Underwood et al., 2017). Our study demonstrates that ALAN can impact the physiology of two coral species from the Gulf of Aqaba/Eilat. The two coral species tested in this study both showed sensitivity to POL, exhibiting lower performances in the ETR, increases in the oxidative stress condition, changes in symbiotic algae density, and chlorophyll concentrations. Our results emphasize the different responses observed in both coral species, where *P. damicornis* experienced more sensitivity in comparison to *A. erystoma*. As for the light treatment, although the monochromatic LED light had an impact on coral physiology including the White LED, while the Yellow light had a less pronounced affect. Although the use of artificial lighting at night has provided obvious benefits to humankind, it has also disrupted natural daily, seasonal, and lunar light cycles as experienced by a diversity of organisms. Hence, it has altered cues for the timings of many biological activities. The ability of organisms to rapidly adapt to the introduction of ALAN through behavioral, genetic, or epigenetic changes is likely to be far more limited than for climate warming due to the unprecedented nature of this change (Swaddle et al., 2015). Therefore, it is important to assess and manage the impact of POL in marine coastal zones to prevent a degradation of marine ecological systems, like coral reefs, found near urban areas.

## CONFLICT OF INTEREST

The authors declare that there is no conflict of interest.

## References

[gcb14795-bib-0001] Allen, J. F. (2003). Cyclic, pseudocyclic and noncyclic photophosphorylation: New links in the chain. Trends in Plant Science, 8(1), 15–19. 10.1016/S1360-1385(02)00006-7 12523995

[gcb14795-bib-0002] Bentley, M. G. , Olive, P. J. W. , & Last, K. (2001). Sexual satellites, moonlight and the nuptial dances of worms: The influence of the moon on the reproduction of marine animals In Barbieri C. & Rampazzi F. (Eds.), Earth‐Moon relationships (pp. 67–84). Dordrecht, the Netherlands: Springer Retrieved from http://link.springer.com/10.1007/978-94-010-0800-6_7

[gcb14795-bib-0003] Bird, B. L. , Branch, L. C. , & Miller, D. L. (2004). Effects of coastal lighting on foraging behavior of beach mice. Conservation Biology, 18(5), 1435–1439. 10.1111/j.1523-1739.2004.00349.x

[gcb14795-bib-0004] Bradford, M. M. (1976). A rapid and sensitive method for the quantitation of microgram quantities of protein utilizing the principle of protein‐dye binding. Analytical Biochemistry, 72, 248–254. 10.1006/abio.1976.9999 942051

[gcb14795-bib-0005] Cinzano, P. , Falchi, F. , & Elvidge, C. D. (2001). The first world atlas of the artificial night sky brightness. Monthly Notices of the Royal Astronomical Society, 328(3), 689–707. 10.1046/j.1365-8711.2001.04882.x

[gcb14795-bib-0006] Cunning, R. , & Baker, A. C. (2013). Excess algal symbionts increase the susceptibility of reef corals to bleaching. Nature Climate Change, 3(3), 259–262. 10.1038/nclimate1711

[gcb14795-bib-0007] Davies, T. W. , Bennie, J. , Cruse, D. , Blumgart, D. , Inger, R. , & Gaston, K. J. (2017). Multiple night‐time light‐emitting diode lighting strategies impact grassland invertebrate assemblages. Global Change Biology, 23(7), 2641–2648. 10.1111/gcb.13615 28139040

[gcb14795-bib-0008] Davies, T. W. , Duffy, J. P. , Bennie, J. , & Gaston, K. J. (2014). The nature, extent, and ecological implications of marine light pollution. Frontiers in Ecology and the Environment, 12(6), 347–355. 10.1890/130281

[gcb14795-bib-0009] de Aguiar, R. B. , Dickel, O. E. , Cunha, R. W. , Monserrat, J. M. , Barros, D. M. , & Martinez, P. E. (2008). Estradiol valerate and tibolone: Effects upon brain oxidative stress and blood biochemistry during aging in female rats. Biogerontology, 9(5), 285–298. 10.1007/s10522-008-9137-7 18386154

[gcb14795-bib-0010] De'ath, G. , Fabricius, K. E. , Sweatman, H. , & Puotinen, M. (2012). The 27‐year decline of coral cover on the great barrier reef and its causes. Proceedings of the National Academy of Sciences of the United States of America, 109(44), 17995–17999. 10.1073/pnas.1208909109 23027961 PMC3497744

[gcb14795-bib-0011] Downs, C. A. , Ostrander, G. K. , Rougee, L. , Rongo, T. , Knutson, S. , Williams, D. E. , … Richmond, R. H. (2012). The use of cellular diagnostics for identifying sub‐lethal stress in reef corals. Ecotoxicology, 21(3), 768–782. 10.1007/s10646-011-0837-4 22215560

[gcb14795-bib-0012] Duarte, C. , Quintanilla‐Ahumada, D. , Anguita, C. , Manríquez, P. H. , Widdicombe, S. , Pulgar, J. , … Quijón, P. A. (2019). Artificial light pollution at night (ALAN) disrupts the distribution and circadian rhythm of a sandy beach isopod. Environmental Pollution, 248, 565–573. 10.1016/j.envpol.2019.02.037 30831353

[gcb14795-bib-0013] Falchi, F. , Cinzano, P. , Duriscoe, D. , Kyba, C. C. M. , Elvidge, C. D. , Baugh, K. , … Furgoni, R. (2016). The new world atlas of artificial night sky brightness. Science Advances, 2(6), e1600377 10.1126/sciadv.1600377 27386582 PMC4928945

[gcb14795-bib-0014] Fitt, W. K. , Spero, H. J. , Halas, J. , White, M. W. , & Porter, J. W. (1993). Recovery of the coral montastrea annularis in the florida keys after the 1987 Caribbean? Bleaching event? Coral Reefs, 12(2), 57–64. 10.1007/BF00302102

[gcb14795-bib-0015] Gardner, S. G. , Raina, J.‐B. , Nitschke, M. R. , Nielsen, D. A. , Stat, M. , Motti, C. A. , … Petrou, K. (2017). A multi‐trait systems approach reveals a response cascade to bleaching in corals. BMC Biology, 15(1), 117 Retrieved from https://bmcbiol.biomedcentral.com/articles/10.1186/s12915-017-0459-2 29216891 10.1186/s12915-017-0459-2 PMC5719617

[gcb14795-bib-0016] Garrett, J. K. , Donald, P. F. , & Gaston, K. J. (2019). Skyglow extends into the world's Key Biodiversity Areas. Animal Conservation. 10.1111/acv.12480

[gcb14795-bib-0017] Gaston, K. J. , Bennie, J. , Davies, T. W. , & Hopkins, J. (2013). The ecological impacts of nighttime light pollution: A mechanistic appraisal: Nighttime light pollution. Biological Reviews, 88(4), 912–927. 10.1111/brv.12036 23565807

[gcb14795-bib-0018] Gaston, K. J. , Davies, T. W. , Nedelec, S. L. , & Holt, L. A. (2017). Impacts of artificial light at night on biological timings. Annual Review of Ecology, Evolution, and Systematics, 48(1), 49–68. 10.1146/annurev-ecolsys-110316-022745

[gcb14795-bib-0019] Heber, U. , & Walker, D. (1992). Concerning a dual function of coupled cyclic electron transport in leaves. Plant Physiology, 100(4), 1621–1626. 10.1104/pp.100.4.1621 16653176 PMC1075843

[gcb14795-bib-0020] Higuchi, T. , Agostini, S. , Casareto, B. E. , Suzuki, Y. , & Yuyama, I. (2016). The northern limit of corals of the genus Acropora in temperate zones is determined by their resilience to cold bleaching. Scientific Reports, 5(1). Retrieved from http://www.nature.com/articles/srep18467 10.1038/srep18467 PMC4683436 26680690

[gcb14795-bib-0021] Hoegh‐Guldberg, O. (2014). Coral Reef Sustainability through Adaptation: Glimmer of Hope or Persistent Mirage? Current Opinion in Environmental Sustainability, 7, 127–133. 10.1016/j.cosust.2014.01.005

[gcb14795-bib-0022] Hoogenboom, M. O. , Campbell, D. A. , Beraud, E. , DeZeeuw, K. , & Ferrier‐Pagès, C. (2012). Effects of light, food availability and temperature stress on the function of photosystem II and photosystem I of coral symbionts. PLoS ONE, 7(1), e30167 10.1371/journal.pone.0030167 22253915 PMC3258252

[gcb14795-bib-0023] Huang, D. , Ou, B. , & Prior, R. L. (2005). The Chemistry behind antioxidant capacity assays. Journal of Agricultural and Food Chemistry, 53(6), 1841–1856. 10.1021/jf030723c 15769103

[gcb14795-bib-0024] Jeffrey, S. W. , & Humphrey, G. F. (1975). New spectrophotometric equations for determining chlorophylls a, b, C1 and C2 in higher plants, algae and natural phytoplankton. Biochemie Und Physiologie Der Pflanzen, 167(2), 191–194. 10.1016/S0015-3796(17)30778-3

[gcb14795-bib-0025] Johannes, R. E. , & Wiebe, W. J. (1970). Method for determination of coral tissue biomass and composition. Limnology and Oceanography, 15(5), 822–824. 10.4319/lo.1970.15.5.0822

[gcb14795-bib-0026] Johnson, G. N. (2004). Cyclic electron transport in C3 plants: Fact or artefact? Journal of Experimental Botany, 56(411), 407–416. 10.1093/jxb/eri106 15647314

[gcb14795-bib-0027] Jombart, T. (2008). adegenet: A R package for the multivariate analysis of genetic markers. Bioinformatics, 24(11), 1403–1405. 10.1093/bioinformatics/btn129 18397895

[gcb14795-bib-0028] Kaniewska, P. , Alon, S. , Karako‐Lampert, S. , Hoegh‐Guldberg, O. , & Levy, O. (2015). Signaling cascades and the importance of moonlight in coral broadcast mass spawning. eLife, 4, e09991 10.7554/elife.09991 26668113 PMC4721961

[gcb14795-bib-0029] Karako‐Lampert, S. , Katcoff, D. J. , Achituv, Y. , Dubinsky, Z. , & Stambler, N. (2004). Do clades of symbiotic dinoflagellates in scleractinian corals of the Gulf of Eilat (Red Sea) differ from those of other coral reefs? Journal of Experimental Marine Biology and Ecology, 311(2), 301–314. 10.1016/j.jembe.2004.05.015

[gcb14795-bib-0030] LaJeunesse, T. C. , Parkinson, J. E. , Gabrielson, P. W. , Jeong, H. J. , Reimer, J. D. , Voolstra, C. R. , & Santos, S. R. (2018). Systematic revision of Symbiodiniaceae highlights the antiquity and diversity of coral endosymbionts. Current Biology, 28(16), 2570–2580.e6. 10.1016/j.cub.2018.07.008 30100341

[gcb14795-bib-0031] Lesser, M. P. (2006). Oxidative stress in marine environments: Biochemistry and physiological ecology. Annual Review of Physiology, 68(1), 253–278. 10.1146/annurev.physiol.68.040104.110001 16460273

[gcb14795-bib-0032] Levy, O. , Achituv, Y. , Yacobi, Y. Z. , Stambler, N. , & Dubinsky, Z. (2006). The impact of spectral composition and light periodicity on the activity of two antioxidant enzymes (SOD and CAT) in the coral *Favia favus* . Journal of Experimental Marine Biology and Ecology, 328(1), 35–46. 10.1016/j.jembe.2005.06.018

[gcb14795-bib-0033] Levy, O. , Dubinsky, Z. , Schneider, K. , Achituv, Y. , Zakai, D. , & Gorbunov, M. Y. (2004). Diurnal hysteresis in coral photosynthesis. Marine Ecology Progress Series, 268, 105–117. 10.3354/meps268105

[gcb14795-bib-0034] Levy, O. , Mizrahi, L. , Chadwick‐Furman, N. E. , & Achituv, Y. (2001). Factors controlling the expansion behavior of *Favia favus* (Cnidaria: Scleractinia): Effects of light, flow, and planktonic prey. The Biological Bulletin, 200(2), 118–126. 10.2307/1543305 11341573

[gcb14795-bib-0035] Longcore, T. , & Rich, C. (2004). Ecological light pollution. Frontiers in Ecology and the Environment, 2(4), 191–198. 10.1890/1540-9295(2004)002[0191:ELP]2.0.CO;2

[gcb14795-bib-0036] Luarte, T. , Bonta, C. C. , Silva‐Rodriguez, E. A. , Quijón, P. A. , Miranda, C. , Farias, A. A. , & Duarte, C. (2016). Light pollution reduces activity, food consumption and growth rates in a sandy beach invertebrate. Environmental Pollution, 218, 1147–1153. 10.1016/j.envpol.2016.08.068 27589894

[gcb14795-bib-0037] Moberg, F. , & Folke, C. (1999). Ecological goods and services of coral reef ecosystems. Ecological Economics, 29(2), 215–233. 10.1016/S0921-8009(99)00009-9

[gcb14795-bib-0038] Muscatine, L. , Falkowski, P. G. , Dubinsky, Z. , Cook, P. A. , & McCloskey, L. R. (1989). The effect of external nutrient resources on the population dynamics of zooxanthellae in a reef coral. Proceedings of the Royal Society B: Biological Sciences, 236(1284), 311–324. 10.1098/rspb.1989.0025

[gcb14795-bib-0039] Muscatine, L. , Goiran, C. , Land, L. , Jaubert, J. , Cuif, J.‐P. , & Allemand, D. (2005). Stable isotopes (13C and 15N) of organic matrix from coral skeleton. Proceedings of the National Academy of Sciences of the United States of America, 102(5), 1525–1530. 10.1073/pnas.0408921102 15671164 PMC547863

[gcb14795-bib-0040] Nesa, B. , & Hidaka, M. (2009). High zooxanthella density shortens the survival time of coral cell aggregates under thermal stress. Journal of Experimental Marine Biology and Ecology, 368(1), 81–87. 10.1016/j.jembe.2008.10.018

[gcb14795-bib-0041] Nicholls, R. J. (1995). Coastal megacities and climate change. GeoJournal, 37(3), 369–379. 10.1007/BF00814018

[gcb14795-bib-0042] Oakes, K. D. , & Van Der Kraak, G. J. (2003). Utility of the TBARS assay in detecting oxidative stress in white sucker (*Catostomus commersoni*) populations exposed to pulp mill effluent. Aquatic Toxicology (Amsterdam, Netherlands), 63(4), 447–463. 10.1016/S0166-445X(02)00204-7 12758008

[gcb14795-bib-0043] Ralph, P. J. , & Gademann, R. (2005). Rapid light curves: A powerful tool to assess photosynthetic activity. Aquatic Botany, 82(3), 222–237. 10.1016/j.aquabot.2005.02.006

[gcb14795-bib-0044] Richier, S. , Rodriguez‐Lanetty, M. , Schnitzler, C. E. , & Weis, V. M. (2008). Response of the symbiotic cnidarian *Anthopleura elegantissima* transcriptome to temperature and UV increase. Comparative Biochemistry and Physiology Part D: Genomics and Proteomics, 3(4), 283–289. 10.1016/j.cbd.2008.08.001 20494848

[gcb14795-bib-0045] Richter, M. , Rühle, W. , & Wild, A. (1990). Studies on the mechanism of photosystem II photoinhibition II. The involvement of toxic oxygen species. Photosynthesis Research, 24, 237–243. 24420076 10.1007/BF00032311

[gcb14795-bib-0046] Sebens, K. P. (1994). Biodiversity of coral reefs: What are we losing and why? American Zoologist, 34(1), 115–133. 10.1093/icb/34.1.115

[gcb14795-bib-0047] Sebens, K. P. , & DeRiemer, K. (1977). Diel cycles of expansion and contraction in coral reef anthozoans. Marine Biology, 43(3), 247–256. 10.1007/BF00402317

[gcb14795-bib-0048] Stimson, J. , & Kinzie, R. A. (1991). The temporal pattern and rate of release of zooxanthellae from the reef coral *Pocillopora damicornis* (Linnaeus) under nitrogen‐enrichment and control conditions. Journal of Experimental Marine Biology and Ecology, 153(1), 63–74. 10.1016/S0022-0981(05)80006-1

[gcb14795-bib-0049] Swaddle, J. P. , Francis, C. D. , Barber, J. R. , Cooper, C. B. , Kyba, C. C. M. , Dominoni, D. M. , … Longcore, T. (2015). A framework to assess evolutionary responses to anthropogenic light and sound. Trends in Ecology & Evolution, 30(9), 550–560. 10.1016/j.tree.2015.06.009 26169593

[gcb14795-bib-0050] Tamir, R. , Lerner, A. , Haspel, C. , Dubinsky, Z. , & Iluz, D. (2017). The spectral and spatial distribution of light pollution in the waters of the northern Gulf of Aqaba (Eilat). Scientific Reports, 7, 42329 10.1038/srep42329 28186138 PMC5301253

[gcb14795-bib-0051] Underwood, C. N. , Davies, T. W. , & Queirós, A. M. (2017). Artificial light at night alters trophic interactions of intertidal invertebrates. Journal of Animal Ecology, 86(4), 781–789. 10.1111/1365-2656.12670 28452048

[gcb14795-bib-0052] Weis, V. M. , Davy, S. K. , Hoegh‐Guldberg, O. , Rodriguez‐Lanetty, M. , & Pringle, J. R. (2008). Cell biology in model systems as the key to understanding corals. Trends in Ecology & Evolution, 23(7), 369–376. 10.1016/j.tree.2008.03.004 18501991

[gcb14795-bib-0053] Yahel, R. , Yahel, G. , Berman, T. , Jaffe, J. S. , & Genin, A. (2005). Diel pattern with abrupt crepuscular changes of zooplankton over a coral reef. Limnology and Oceanography, 50(3), 930–944. 10.4319/lo.2005.50.3.0930

