# Supplementary material for: Red Sea corals under Artificial Light Pollution at Night (ALAN) undergo oxidative stress and photosynthetic impairment
Source: Glob Chang Biol. 2019 Sep 11;25(12):4194–207. doi: 10.1111/gcb.14795 (PMC6900201; doi:10.1111/gcb.14795)
Supplement: Supplementary file 1 [file GCB-25-4194-s001.docx]

**Supplementary Figures and Tables**

**Red Sea corals under Artificial Light at Night (ALAN) undergo oxidative stress and photosynthetic impairment**

**Running Title: ALAN impact on coral physiology**

Inbal Ayalon^1,2,3*^, Laura F. de Barros Marangoni^1^, Jennifer I.C. Benichou^1^

Dror Avisar^3^ and Levy Oren^1*^

^1^Mina and Everard Goodman Faculty of Life Sciences, Bar-Ilan University, Ramat Gan 52900, Israel ^2^Israel The H. Steinitz Marine Biology Laboratory, The Interuniversity Institute for Marine Sciences of Eilat, P.O. Box 469, Eilat 88103, Israel ^3^Porter School of the Environment and Earth Sciences, Faculty of Exact Sciences, Tel Aviv University, 39040, Israel

**Keywords:** Corals, light pollution, ALAN, physiology, ROS, photosynthesis

Corresponding authors e-mail: inbalaya@gmail.com & oren.levy@biu.ac.il

A

B

**Figure S1**- (A) Long term experiment, LEED lamp- Light spectrum, measured temperature and light intensity during day time. (B) Short term experiment LEED’s lamps- Light spectrum (Blue, White and Yellow), measured temperature and light intensity during day time.

**Figure S2** (Related to Fig. 1) – Raw data, relationship between irradiance and ETR in the corals *A. eurystoma* (A-B) and *P.* *damicornis* (C-D) under different light conditions [Ambient and Light polluted (POL)] after different periods of exposure [Exp 1: 40 (T2) and 120 (T6) days]. Mean ETR +/- SEM for each irradiance levels.

**Figure S3** (Related to Fig. 2) – Raw data, relationship between irradiance and ETR in the corals *A. eurystoma* (A-D) and *P. damicornis* (E-H) exposed to different monochromatic light conditions [Ambient, Blue, White and Yellow lights] at different daylight hours (5 and 11AM) and times of exposure [Exp 2: 10 (T1) and 20 (T2) days]. Mean ETR +/- SEM for each irradiance levels.

**Figure S4:** Effect of different light conditions [Ambient, Blue, White and Yellow] in the coral *A. eurystoma* in several physiological parameters. Data is expressed as mean ± SE (n = 4-6). Different lowercase letters indicate significantly different mean values (p < 0.05).

**Figure S5:** Effect of different light conditions [Ambient, Blue, White and Yellow] in the coral *P. damicornis* in several physiological parameters. Data is expressed as mean ± SE (n = 4-6). Different lowercase letters indicate significantly different mean values (p < 0.05).

**Supplementary Table S1**

Summary of RLC parameters derived from figure 1 after 40 days (T2) of exposure and at the end of the experiment (T6, 120 days), representing maximal photochemical efficiency; rETRmax, relative maximum electron transport rate; α, initial photosynthetic rate under light-limited conditions; Ik, compensation point; and Im maximum saturating irradiance.

|  | ***A. eurystoma* AMB T2** | ***A. eurystoma* POL T2** | ***A. eurystoma* AMB T6** | ***A. eurystoma* POL T6** | ***P. damicornis* AMB T2** | ***P. damicornis* POL T2** | ***P. damicornis* AMB T6** | ***P. damicornis* POL T6** |
| --- | --- | --- | --- | --- | --- | --- | --- | --- |
|  |  |  |  |  |  |  |  |  |
| **rETR_max_** | 20.55 | 18.17 | 26.49 | 21.30 | 26.79 | 21.07 | 28.16 | 22.04 |
| **I_m_** | 219.03 | 215.95 | 287.18 | 221.30 | 289.95 | 246.65 | 315.50 | 259.44 |
| **I_k_** | 80.58 | 79.44 | 95.87 | 76.30 | 106.66 | 81.14 | 116.06 | 89.90 |
| **α** | 0.26 | 0.23 | 0.28 | 0.28 | 0.25 | 0.26 | 0.24 | 0.25 |

**Supplementary Table S2**

Summary of RLC parameters derived from figure 2 for *A. eurystoma* under the different Blue, White, Yellow and AMB LED’s at two different hours 05:00 am and 11 am, Exp 2: after 10 (T1) and 20 (T2) days representing maximal photochemical efficiency; rETRmax, relative maximum electron transport rate; α, initial photosynthetic rate under light-limited conditions; Ik, compensation point; and Im maximum saturating irradiance.

| ***A. eurystoma* T-1 05:00** | **AMB** | **BLUE** | **WHITE** | **YELLOW** | **T-1 11:00** | **AMB** | **BLUE** | **WHITE** | **YELLOW** |
| --- | --- | --- | --- | --- | --- | --- | --- | --- | --- |
|  |  |  |  |  |  |  |  |  |  |
| **rETR_max_** | 17.07 | 17.65 | 17.39 | 19.34 |  | 25.63 | 19.80 | 22.51 | 26.46 |
| **I_m_** | 221.03 | 267.36 | 215.32 | 229.13 |  | 301.81 | 282.70 | 264.89 | 303.52 |
| **I_k_** | 60.09 | 55.82 | 56.88 | 61.59 |  | 95.20 | 64.00 | 81.96 | 104.69 |
| **α** | 0.28 | 0.32 | 0.31 | 0.31 |  | 0.27 | 0.31 | 0.27 | 0.25 |
| **T-2 05:00** | **AMB** | **BLUE** | **WHITE** | **YELLOW** | **T-2 11:00** | **AMB** | **BLUE** | **WHITE** | **YELLOW** |
|  |  |  |  |  |  |  |  |  |  |
| **rETR_max_** | 25.85 | 19.44 | 17.18 | 20.53 |  | 28.60 | 23.93 | 21.80 | 22.82 |
| **I_m_** | 277.73 | 261.78 | 210.91 | 242.43 |  | 327.06 | 267.63 | 245.40 | 261.47 |
| **I_k_** | 88.82 | 67.53 | 57.94 | 79.10 |  | 96.95 | 80.29 | 71.34 | 85.36 |
| **α** | 0.29 | 0.29 | 0.30 | 0.26 |  | 0.30 | 0.30 | 0.31 | 0.27 |

**Supplementary Table S3**

Summary of RLC parameters derived from figure 3 for *P. damicornis* under the different monochromatic LED’s at two different hours 05:00 am and 11 am, Exp 2: after 10 (T1) and 20 (T2) days representing maximal photochemical efficiency; rETRmax, relative maximum electron transport rate; α, initial photosynthetic rate under light-limited conditions; Ik, compensation point; and Im maximum saturating irradiance.

| ***P. damicornis* T-1 05:00** | **AMB** | **BLUE** | **WHITE** | **YELLOW** | **T-1 11:00** | **AMB** | **BLUE** | **WHITE** | **YELLOW** |
| --- | --- | --- | --- | --- | --- | --- | --- | --- | --- |
|  |  |  |  |  |  |  |  |  |  |
| **rETR_max_** | 28.54 | 20.06 | 20.71 | 24.66 |  | 31.62 | 25.16 | 25.96 | 32.55 |
| **I_m_** | 276.28 | 357.75 | 258.51 | 329.26 |  | 301.51 | 273.89 | 305.40 | 310.64 |
| **I_k_** | 81.70 | 65.76 | 51.60 | 66.15 |  | 107.75 | 78.93 | 93.81 | 96.88 |
| **α** | 0.35 | 0.31 | 0.40 | 0.37 |  | 0.29 | 0.32 | 0.28 | 0.34 |
| **T-2 05:00** | **AMB** | **BLUE** | **WHITE** | **YELLOW** | **T-2 11:00** | **AMB** | **BLUE** | **WHITE** | **YELLOW** |
|  |  |  |  |  |  |  |  |  |  |
| **rETR_max_** | 24.60 | 22.75 | 21.05 | 21.44 |  | 36.30 | 23.65 | 23.33 | 23.94 |
| **I_m_** | 295.10 | 282.03 | 237.98 | 233.46 |  | 417.79 | 281.88 | 263.56 | 295.21 |
| **I_k_** | 85.60 | 92.80 | 70.32 | 70.05 |  | 136.53 | 83.97 | 74.79 | 84.57 |
| **α** | 0.29 | 0.25 | 0.30 | 0.31 |  | 0.27 | 0.28 | 0.31 | 0.28 |
